# Body Water Content and Morphological Characteristics Modify Bioimpedance Vector Patterns in Volleyball, Soccer, and Rugby Players

**DOI:** 10.3390/ijerph17186604

**Published:** 2020-09-10

**Authors:** Francesco Campa, Analiza M. Silva, Catarina N. Matias, Cristina P. Monteiro, Antonio Paoli, João Pedro Nunes, Jacopo Talluri, Henry Lukaski, Stefania Toselli

**Affiliations:** 1 Department for Life Quality Studies, University of Bologna, 47921 Rimini, Italy; 2 Exercise and Health Laboratory, CIPER, Faculdade Motricidade Humana, Universidade de Lisboa, 1499-002 Cruz Quebrada, Portugal; analiza@fmh.ulisboa.pt (A.M.S.); cmatias@fmh.ulisboa.pt (C.N.M.); cmonteiro@fmh.ulisboa.pt (C.P.M.); 3 Department of Biomedical Science, University of Padova, 35100 Padova, Italy; antonio.paoli@unipd.it; 4 Metabolism, Nutrition, and Exercise Laboratory, Physical Education and Sports Center, Londrina State University, 86057 Londrina, Brazil; joaonunes.jpn@hotmail.com; 5 Department of clinical research and development, Akern Ltd., 56121 Pisa, Italy; jacopo.talluri@akern.com; 6 Department of Kinesiology and Public Health Education, Hyslop Sports Center, University of North Dakota, Grand Forks, ND 58202, USA; henry.lukaski@und.edu; 7 Department of Biomedical and Neuromotor Sciences, University of Bologna, 40126 Bologna, Italy; stefania.toselli@unibo.it

**Keywords:** body composition, BIVA, phase angle, R-Xc graph, somatotype, total body water, vector length

## Abstract

**Background:** Bioimpedance vector analysis (BIVA) is a widely used method based on the interpretation of raw bioimpedance parameters to evaluate body composition and cellular health in athletes. However, several variables contribute to influencing BIVA patterns by militating against an optimal interpretation of the data. This study aims to explore the association of morphological characteristics with bioelectrical properties in volleyball, soccer, and rugby players. **Methods:** 164 athletes belonging to professional teams (age 26.2 ± 4.4 yrs; body mass index (BMI) 25.4 ± 2.4 kg/m^2^) underwent bioimpedance and anthropometric measurements. Bioelectric resistance (R) and reactance (Xc) were standardized for the athlete’s height and used to plot the vector in the R-Xc graph according to the BIVA approach. Total body water (TBW), phase angle (PhA), and somatotype were determined from bioelectrical and anthropometric data. **Results:** No significant difference (*p* > 0.05) for age and for age at the start of competition among the athletes was found. Athletes divided into groups of TBW limited by quartiles showed significant differences in the mean vector position in the R-Xc graph (*p* < 0.001), where a higher content of body fluids resulted in a shorter vector and lower positioning in the graph. Furthermore, six categories of somatotypes were identified, and the results of bivariate and partial correlation analysis highlighted a direct association between PhA and mesomorphy (*r* = 0.401, *p* < 0.001) while showing an inverse correlation with ectomorphy (r = −0.416, p < 0.001), even adjusted for age. On the contrary, no association was observed between PhA and endomorphy (*r* = 0.100, *p* = 0.471). **Conclusions:** Body fluid content affects the vector length in the R-Xc graph. In addition, the lateral displacement of the vector, which determines the PhA, can be modified by the morphological characteristics of the athlete. In particular, higher PhA values are observed in subjects with a high mesomorphic component, whereas lower values are found when ectomorphy is dominant.

## 1. Introduction

In recent years, the bioimpedance vector analysis (BIVA) has been widely used in the sports field for the assessment of body composition and cellular health in athletes [1]. This is because BIVA is not subject to errors related to prediction equations since it interprets the raw bioimpedance values [resistance (R) and reactance (Xc)], and it is an easy to use and non-invasive method. Its application is based on the bivariate interpretation of R and Xc standardized for height on a graph. Vector displacements identify increases or losses in total body water (TBW) or in the ratio between intra (ICW) and extracellular (ECW) fluids for which increases or decreases correspond to shifts to the left or to the right of the R-Xc graph, respectively [2]. In addition, it is possible to obtain an immediate analysis of the subject’s body composition by comparing the vector position with tolerance ellipses built from the data of the reference population [3,4,5,6].

Recent studies have focused on evaluating factors influencing the vector position of athletes, including maturity status, [7,8,9,10] dehydration [11,12,13,14], and fitness level [15]. Since the bioelectric properties of body tissues depend on body fluids and cells’ membrane integrity [16], the main determinant of the vector position in the R-Xc graph is the TBW and the distribution of the fluids among the two compartments (ICW and ECW). In fact, the vector length is inversely proportional to TBW, while the lateral displacements of the vector are directly correlated with the ICW/ECW ratio [17,18,19,20]. In addition to these body composition variables, Campa et al. [21] have recently suggested that the somatotype also influences vector position in the R-Xc graph, where athletes with higher mesomorphic and endomorphic components are positioned more to the lower-left than athletes with a dominant ectomorphy. However, as the content of total body fluids is greatly associated with the vector, correct discrimination in the R-Xc graph based on somatotype categories may be compromised. Indeed, athletes with high body weight can still be located at the bottom of the graph regardless of their morphology.

The bioelectric values and the vector position reflect body composition; in particular, the phase angle (PhA), obtained as the arctangent of Xc/R, correctly mirrors the ICW/ECW ratio [17,18,19,20]. In fact, athletes with a high PhA are positioned to the left portion in the R-Xc graph [21,22], and increases in muscle mass, body cell mass, and, therefore, ICW lead to vector shifts further to the left over time [23,24]. A high muscularity is observed in subjects with a high PhA or in those whose somatotype shows a dominant mesomorphic component [21,22], as mesomorphy characterizes skeletal muscle features [25]; moreover, both PhA and somatotype can be modified with nutrition and exercise [23,26]. However, to the best of our knowledge, no study has explored the associations between somatotype and PhA, while analyzing the influence of morphology on the vector position for similar TBW values in athletes.

Therefore, this study aimed to analyze the associations of morphological characteristics with bioelectrical properties using the BIVA approach, according to different levels of body water content in male volleyball, soccer, and rugby players. Our hypothesis was that PhA was associated with morphological characteristics in the athletes.

## 2. Methods

### 2.1. Subjects

This was a cross-sectional observational study conducted in 164 athletes engaged in 7 professional Italian teams participating in Series A2, Series B, and Series A divisions of volleyball, soccer, and rugby, respectively (age 26.2 ± 4.4 yrs; body mass index (BMI) 25.4 ± 2.4 kg/m^2^; age at start competition 14.2 ± 1.3 yrs). The following inclusion criteria were used: (i) A minimum of 10 h of training per week; (ii) tested negative for performance-enhancing drugs; and (iii) not taking any medication. The athletes were tested in the morning (9:00 AM) in the facilities of the teams. All measurements were performed under resting conditions in the second off-season period. All participants gave informed consent after receiving a detailed description of the study procedures. The project was conducted in accordance with the guidelines of the Declaration of Helsinki and was approved by the local Bioethics Committee of the University of Bologna. (Ethical Approval Code: 25027).

### 2.2. Procedures

All athletes were tested to ensure a well-hydrated state using the urine specific gravity test (refractometer Urisys 1100; Roche Diagnostics), according to Armstrong et al. [27]. A urine specific gravity value < 1.022 for the first urine was used to identify an euhydration state.

The anthropometric traits were body mass, height, humerus and femur breadths, contracted arm and calf girths, and 4-skinfold thicknesses (triceps, subscapular, supraspinal, and medial calf). All anthropometric measurements were taken by a certified anthropometrist according to standard methods in the literature [28], whose technical error was 5% and 1.5% for skinfolds and all other measurements, respectively. Height was recorded to the nearest 0.1 cm using a stadiometer (Raven Equipment Ltd., Great Donmow, UK) and body mass was measured to the nearest 0.1 kg using a high-precision mechanical scale (Seca, Basel, Switzerland). BMI was calculated as the ratio of body weight to height squared (kg/m^2^). Girths were taken to the nearest 0.1 cm using a tape measure (GMP, Zürich, Switzerland). Breadths were measured to the nearest 0.1 cm using a sliding caliper (GMP, Zürich, Switzerland). Skinfold thicknesses were measured to the nearest 0.1 mm using a Lange skinfold caliper (Beta technology Inc., Cambridge, MD, USA).

Bioimpedance analysis (BIA) was performed by a phase-sensitive single-frequency bioimpedance analyzer (101 Anniversary, Akern, Florence, Italy), which applied an alternating current of 400 microamperes at 50 kHz. Vector length (VL) was calculated as (adjusted R^2^ + adjusted Xc^2^)^0.5^ and PhA as the arctangent of Xc/R × 180/π. BIVA was applied to normalize V, R, and Xc for height (H) in meters [29]. TBW was calculated from bioimpedance values, according to specific equations developed for athletes using a 4-compartment model as a criterion method [30] then the athletes were divided into quartiles.

Somatotype components were calculated according to the Heath and Carter method [25] as follow:

*Endomorphy* = − 0.7182 + 0.1451 (X) − 0.00068 (X 2) + 0.0000014 (X 3), where X = (sum of triceps, subscapular and supraspinal skinfolds) multiplied by (170.18/H in cm);

*Mesomorphy* = 0.858 × humerus breadth + 0.601 × femur breadth + 0.188 × corrected arm girth + 0.161 × corrected calf girth − H 0.131 + 4.5;

*Ectomorphy* = 0.732 HWR − 28.58, where HWR = (height divided by the cube root of weight).

From the 13 initial proposed categories by Heath and Carter [25], the athletes were grouped in 6 somatotype categories:

- Endomorphic mesomorph (EnM): Mesomorphy is dominant and endomorphy is greater than ectomorphy.
- Balanced mesomorph (BM): Mesomorphy is dominant and endomorphy and ectomorphy are equal.
- Ectomorphic mesomorph (EcM): Mesomorphy is dominant and ectomorphy is greater than endomorphy.
- Mesomorph-ectomorph (M-Ec): Mesomorphy and ectomorphy are equal, and endomorphy is smaller.
- Mesomorphic ectomorph (MEc): Ectomorphy is dominant and mesomorphy is greater than endomorphy.
- Balanced ectomorph (Bec): Ectomorphy is dominant and endomorphy and mesomorphy are equal.

### 2.3. Statistical Analysis

To verify the normality of the data, the Shapiro-Wilk test was applied. The athletes were divided into groups limited by quartiles of TBW and the one-way ANOVA was performed to evaluate the difference in BIVA patterns (PhA and VL/H). When a significant F ratio was obtained, the Bonferroni post hoc test was used to assess the differences between the 4 groups, setting the significance at *p* < 0.008. The two-sample Hotelling’s T^2^ test was used to compare the mean impedance vectors among the athletes grouped according to quartiles of TBW. Bivariate and partial (controlling for age) correlations were performed to evaluate the associations between PhA and the somatotype components. The mean standard deviation was calculated for each variable. Data were analyzed with IBM SPSS Statistics, version 24.0 (IBM Corp., Armonk, NY, USA).

## 3. Results

**Figure 1:**
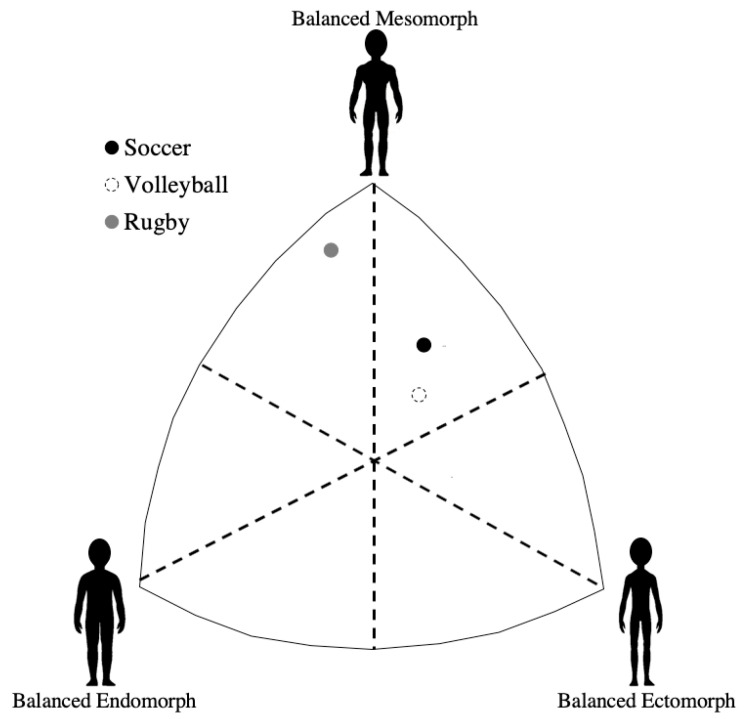
Representation of the athletes’ somatotype.

No significant difference (*p* > 0.05) for age and for age at start of competition among the athletes was found. The soccer, volleyball, and rubgy players showed an average EcM (endomorphy: 1.6 ± 0.3; mesomorphy: 4.7 ± 0.9; ectomorphy: 2.9 ± 0.8), EcM (endomorphy: 2.0 ± 0.7; mesomorphy: 4.0 ± 1.3; ectomorphy: 3.2 ± 1.1), and EnM (endomorphy: 2.1 ± 0.7; mesomorphy: 6.0 ± 1.1; ectomorphy: 0.9 ± 0.3) somatotype, respectively (Figure 1).

**Figure 2:**
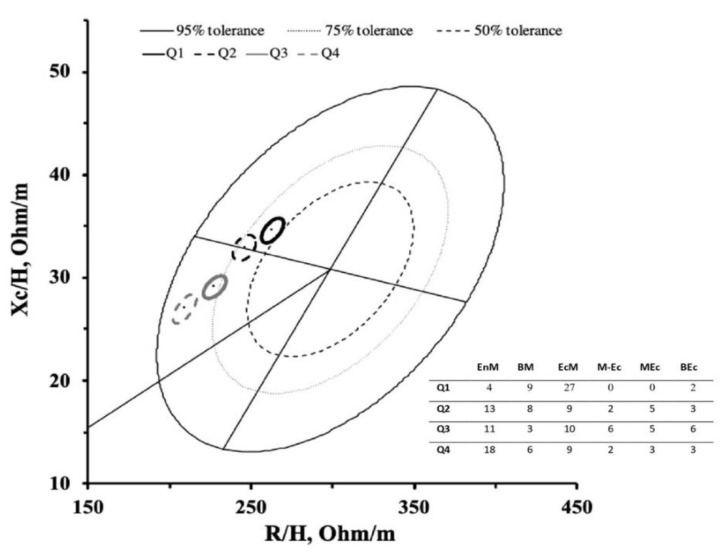
Scattergram of the mean impedance vectors of the athletes divided by the total body water groups plotted on the 50%, 75%, and 95% tolerance ellipses of the healthy male Italian population [31]; on the right side, the absolute frequencies of the somatotype categories for each quartile is shown. EnM = Endomorphic Mesomorph, BM = Balanced Mesomorph, EcM = Ectomorphic Mesomorph, M-Ec = Mesomorph Ectomorph, MEc = Mesomorphic Ectomorph, BEc = Balanced Ectomorph.

Descriptive body fluids and bioelectrical characteristics are presented in Table 1, while the mean impedance vectors of the athletes divided according to quartiles of TBW are shown in Figure 1. Forty-two athletes were included in the first group (Q1) (endomorphy: 1.8 ± 0.6, mesomorphy: 4.4 ± 1.1, ectomorphy: 2.9 ± 1.0), 40 in the second group (Q2) (endomorphy: 2.2 ± 0.7, mesomorphy: 4.6 ± 1.5, ectomorphy 2.8 ± 1.3), 41 in the third group (Q3) (endomorphy: 2.2 ± 0.7, mesomorphy: 4.3 ± 1.4, ectomorphy 2.8 ± 1.3) and 41 in the fourth group (Q4) (endomorphy: 2.4 ± 0.7, mesomorphy: 5.2 ± 1.6, ectomorphy 2.0 ± 1.5). Six somatotype categories were identified, and their absolute frequencies for each group are presented in Figure 2. The results of the two-sample Hotelling t^2^ test showed significant differences between all the groups (Q1 vs. Q2, *t* = 21.1, *p* < 0.001; Q1 vs. Q3, *t* = 105.8, *p* < 0.001; Q1 vs. Q4, *p* < 0.001; *t* = 201.6, *p* < 0.001; Q2 vs. Q3, *t* = 39.4, *p* < 0.001; Q2 vs. Q4, *t* = 98.1, *p* < 0.001; Q3 vs. Q4, *t* = 22.7, *p* < 0.001) indicating that the athletes with higher TBW were positioned to the lower left in the R-Xc graph than those with a lower TBW, as displayed in Figure 2. In addition, significant differences (*p* < 0.008) were found between the 4 groups for VL/H but not for PhA, as reported in Table 2.

**Figure 3:**
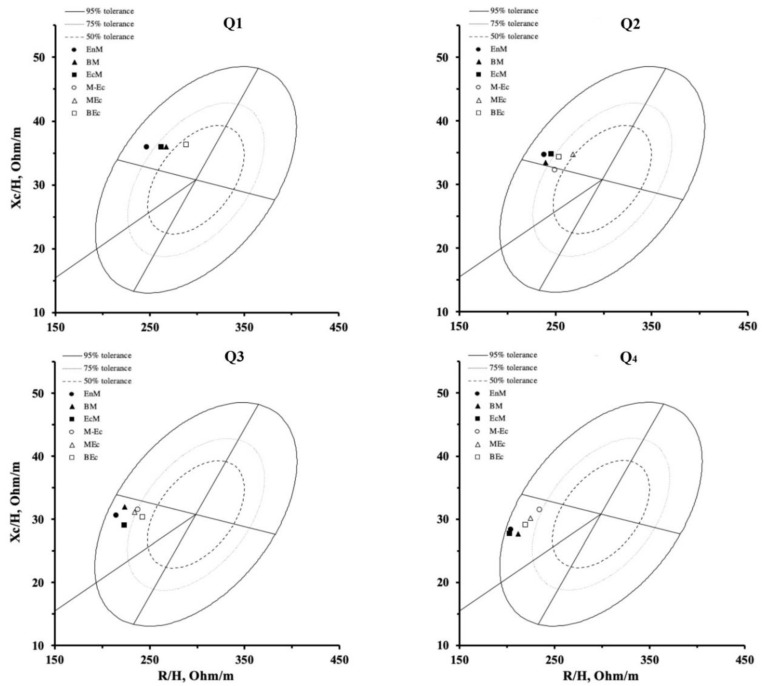
Scattergram of the mean impedance vectors of the athletes are categorized by somatotype and divided according to groups of TBW. EnM = Endomorphic Mesomorph, BM = Balanced Mesomorph, EcM = Ectomorphic Mesomorph, M-Ec = Mesomorph Ectomorph, MEc = Mesomorphic Ectomorph, BEc = Balanced Ectomorph.

**Figure 4:**
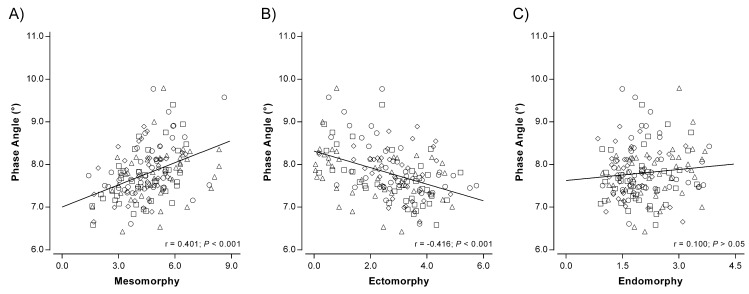
Correlation between phase angle and mesomorphy (**A**), ectomorphy (**B**), and endomorphy (**C**). ^◇^ = subjects from Q1. ^○^ = subjects from Q2. ^□^ = subjects from Q3. ^△^ = subjects from Q4.

Figure 3 illustrates the mean vectors of the athletes subdivided by somatotype in each TBW group. For each TBW group, somatotype categories with a dominant mesomorphy (EnM, BM, and EcM) showed a vector tending to be positioned more to the left than those with a greater ectomorphy (MEc and BEc). Moreover, as displayed in Figure 4, PhA was directly correlated with the mesomorphic component (*r* = 0.401, *p* < 0.001; Panel A) and inversely with the ectomorphic component (*r* = −0.416, *p* < 0.001; Panel B), even when corrected for age (*p* < 0.001). On the contrary, no association was observed between PhA and endomorphy (*r* = 0.100, *p* = 0.471; Panel C).

## 4. Discussion

The aim of this study was to analyze the associations of morphological characteristics with bioelectrical properties in volleyball, soccer, and rugby players. An important finding has emerged from our results regarding the role of body fluids on vector length. In addition, this study has shown, for the first time, the associations between the somatotype components and PhA. As hypothesized, when considering subjects with a similar TBW, the differences in vector position may reflect morphological peculiarities; this was possible to observe due to the data analysis carried out in this study, in which the athletes were divided into separate groups limited by quartiles of TBW.

We observed that body fluids content was a determining factor for vector length, extending the findings of previous research studies [17,20]. In particular, athletes with a higher TBW (Q4) showed a mean vector positioned lower than the other athletes (Figure 2). This is in line with previous studies that observed vector length and its changes to accurately reflect changes in TBW using dilution techniques to assess water and its compartments [17,20,21]. In addition, when the mean vectors for each somatotype category were plotted on the R-Xc graph, it was possible to observe how athletes with a higher mesomorphic component showed a vector tending to be more left in the graph. Conversely, athletes with a dominant ectomorphy presented a vector displacement to the right. In this regard, our results have shown that PhA correlates directly with the mesomorphic component, while an inverse association was observed with the ectomorphic component of the somatotype. These findings are in line with previous investigations that observed in athletes with a higher muscle mass, including bodybuilders, a vector position at the limits of the 95th percentile to the left of the reference ellipses of the normal population [3,22].

The athletes belonging to the six identified somatotype categories were distributed among the four groups of TBW except for the first group where mesomorph ectomorph, and mesomorphic ectomorph athletes, were not present. As a result, it was possible to explore the association between PhA and morphological features. The endomorphic mesomorph, balanced mesomorph, and ectomorphic mesomorph somatotypes are characterized by a dominant mesomorphy, due to a muscular related body shape. On the contrary, mesomorphic ectomorph and balanced ectomorph categories imply a dominant ectomorphy, and therefore, athletes tend to be taller with a lower muscle mass than the other somatotype categories [25]. Rakovi’c et al. [32] showed how mesomorphic features are linked to individual sports that require higher muscle strength, while ectomorphy is predominant in runners [33], especially those involved in long distance. In previous research [21], it was highlighted how R/H and Xc/H were able to discriminate somatotypes. However, if we consider this new and more individual approach, considering the TBW values, probably some of those athletes needed to be revised, since body fluids have a great influence on the vector position. For this reason, the athletes’ somatotypes were analyzed according to groups of body fluids to reduce the differences attributed to TBW, and consequently, to better understand how somatotype is associated with the vector position. Due to this approach, it was possible to observe how the vector position changes based on the morphological features. Indeed, when the athletes were divided into TBW groups, significant differences were found in vector length, but not for PhA, which instead represents the lateral displacement of the vector. This suggests that athletes with a similar PhA could have a greater TBW and, therefore, a different vector position and body composition characteristics. In fact, when comparing the four groups, athletes with a shorter mean vector were those with a higher TBW. A recent literature review on PhA in sports [34] concluded that it was not clear whether PhA differs among athletes engaged in different sports. On the contrary, studies on athletes practicing the same sports, but at different competitive levels, have shown that elite athletes show a higher PhA than those engaged in lower-level categories [4,5,6]. In particular, Micheli et al. [6] suggested that this is due to a lower R/H in relation to Xc/H and reflects a condition of greater muscularity and body cell mass content in the athletes that compete in the higher levels. In this study, it was shown how the interpretation of the vector position in the R-Xc graph overcomes the limits linked to the interpretation of the PhA alone. In this regard, Reis et al. [35] recently showed that the bioimpedance vector position varied in response to changes in the macrocycle training load in swimmers of both sexes. The authors identified an accumulation of fluids and, therefore, a shortening of the vector following a first phase characterized by a high training load, and then a subsequent shift of the vector position to the left side as a result of muscle adaptations that occurred after a recovery period. Similarly, Mascherini et al., in 2014 [24], for the first time studied vector changes over the competitive season in soccer players and showed that PhA decreased during the preparatory phase and then increased near the beginning of the competition. In line with this, Nabuco et al. [36] highlighted a moderate and inverse association between PhA and the values obtained from a fatigue assessment test.

The results of the present study add important and useful information for a correct interpretation of BIVA. The careful evaluation and monitoring of body composition allow the athlete to be predisposed to achieving high peak performance [37]. Through BIVA, it is possible to evaluate the progress in the athlete’s physical condition during the season [38] in response to a training program or a nutritional intervention [37], avoiding incurring decreases in physical performance. This method, in addition to monitoring body fluids, also allows information about other body composition variables at a whole-body level [39]. In fact, it is not always possible to collect the various anthropometric measures that allow the evaluation of the somatotype; in this regard BIVA, if correctly interpreted, can provide important information, minimizing the need for skinfolds and girths collection by a certified anthropometrist. While this method requires further study, especially concerning monitoring the bioimpedance parameters in the short term, the innovation of this study lies in the fact that it provides useful information for the correct interpretation of the vector position in the R-Xc graph, specifically the role of body water as a mediator of the morphological associations with bioelectrical parameters.

Some limitations of this study need to be addressed. First of all, our results are only generalized for male athletes. Secondly, the findings of this study are only applicable to single-frequency BIA equipment. In fact, different results in measuring raw BIA parameters are obtained using devices that work on single- or multi-frequency [40]. On the other hand, the fact that it was ensured that the athletes were in a state of euhydration is a strength of the present work. Future research should study the potential of BIVA patterns as a biomarker of physical condition during the training process, as in specific microcycles. Indeed, as bioimpedance analysis (BIA) equipment provides an easy, simple, and low-cost application, it allows for a frequent assessment to optimize monitoring of the athlete’s physical condition.

## 5. Conclusions

BIVA provides meaningful information on body composition assessment in athletes. This study showed that body fluid content affects the vector length in the R-Xc graph, while the lateral displacement of the vector can be modified by the morphological characteristics of the athlete. In particular, the mesomorphic component of the somatotype is related to higher PhA, an important marker of cellular integrity and overall physical function.

## Figures and Tables

**Table 1:** Descriptive statistics for the athletes divided by total body water quartile [first (Q1), second (Q2), third (Q3), and forth (Q4) quartile] and somatotype.

Variable	Somatotype	Q1 (n = 42)	Q2 (n = 40)	Q3 (n = 41)	Q4 (n = 41)
TBW (L)	EnM	49.2 ± 0.3	52.4 ± 1.5	56.6 ± 1.4	67.5 ± 3.8
	BM	45.9 ± 1.6	51.6 ± 1.6	58.1 ± 1.1	65.1 ± 5.2
	EcM	45.9 ± 3.1	52.5 ± 0.9	56.7 ± 1.3	63.9 ± 4.7
	M-Ec	-	53.7 ± 0.8	56.7 ± 0.9	62.4 ± 1.2
	MEc	-	52.4 ± 0.7	57.7 ± 1.5	61.2 ± 1.2
	BEc	44.2 ± 3.6	51.7 ± 0.9	56.9 ± 1.3	61.6 ± 0.7
	Whole sample	46.1 ± 2.9	52.3 ± 1.3	56.9 ± 1.3	65.2 ± 4.3
R/H (Ohm/m)	EnM	247.0 ± 8.8	238.5 ± 14.1	214.9 ± 18.8	203.9 ± 18.1
	BM	266.8 ± 17.3	238.2 ± 16.9	223.5 ± 6.0	211.6 ± 17.6
	EcM	262.2 ± 15.9	245.5 ± 18.4	223.5 ± 10.8	203.1 ± 12.5
	M-Ec	-	249.2 ± 16.1	237.7 ± 12.8	234.6 ± 29.0
	MEc	-	268.0 ± 9.5	234.5 ± 12.5	224.2 ± 4.9
	BEc	287.8 ± 17.6	253.9 ± 9.5	243.1 ± 17.2	219.6 ± 14.0
	Whole sample	262.9 ± 17.1	245.5 ± 17.3	227.5 ± 17.2	209.0 ± 17.9
Xc/H (Ohm/m)	EnM	35.9 ± 1.4	34.7 ± 3.3	30.7 ± 2.8	28.3 ± 4.0
	BM	36.0 ± 2.7	33.4 ± 2.6	31.9 ± 3.6	27.6 ± 3.9
	EcM	35.9 ± 3.4	34.7 ± 4.1	29.0 ± 2.2	27.7 ± 2.9
	M-Ec	-	32.2 ± 2.3	31.5 ± 2.6	31.4 ± 3.1
	MEc	-	34.7 ± 2.3	31.1 ± 2.6	30.2 ± 2.2
	BEc	36.1 ± 3.3	34.2 ± 1.5	30.4 ± 3.1	29.0 ± 3.8
	Whole sample	35.9 ± 3.0	34.3 ± 3.1	30.5 ± 2.7	28.4 ± 3.5

Note: Data are presented as mean ± SD. TBW = total body water, R/H = resistance standardized for height, Xc/H = reactance standardized for height, EnM = Endomorphic Mesomorph, BM = Balanced Mesomorph, EcM = Ectomorphic Mesomorph, M-Ec = Mesomorph Ectomorph, MEc = Mesomorphic Ectomorph, BEc = Balanced Ectomorph.

**Table 2:** Descriptive statistics for the athletes divided by total body water quartile [first (Q1), second (Q2), third (Q3), and forth (Q4) quartile] and somatotype.

Variable	Somatotype	Q1 (n = 42)	Q2 (n = 40)	Q3 (n = 41)	Q4 (n = 41)	df	F *	*p*
PhA (°)	EnM	8.3 ± 0.1	8.3 ± 0.7	8.1 ± 0.5	8.0 ± 0.7			
	BM	7.7 ± 0.5	7.9 ± 0.6	8.2 ± 1.1	7.4 ± 0.6			
	EcM	7.8 ± 0.6	8.1 ± 0.8	7.4 ± 0.4	7.8 ± 0.5			
	M-Ec	-	7.4 ± 0.5	7.5 ± 0.6	7.7 ± 0.2			
	MEc	-	7.4 ± 0.5	7.6 ± 0.4	7.7 ± 0.4			
	BEc	7.1 ± 0.2	7.7 ± 0.2	7.1 ± 0.3	7.5 ± 0.6			
	Whole sample	7.8 ± 0.5	7.9 ± 0.6	7.7 ± 0.6	7.8 ± 0.6	3	1.8	0.144
VL/H (Ohm/m)	EnM	249.6 ± 8.8	241.1 ± 14.2	217.1 ± 18.9	205.9 ± 18.2			
	BM	269.3 ± 17.3	241.2 ± 16.8	225.8 ± 5.5	213.4 ± 17.9			
	EcM	264.6 ± 16.1	247.9 ± 18.5	225.4 ± 10.9	204.9 ± 12.7			
	M-Ec	-	251.3 ± 16.2	239 ± 12.8	236.6 ± 29.2			
	MEc	-	270.3 ± 9.5	236.6 ± 12.7	226.3 ± 4.2			
	BEc	290.4 ± 17.8	256.2 ± 9.6	244.9 ± 17.4	221.5 ± 18.0			
	Whole sample	265.4 ± 17.1 ^2,3,4^	247.9 ± 17.3 ^1,3,4^	229.5 ± 17.3 ^1,2,4^	210.9 ± 18.1 ^1,2,3^	3	74.7	<0.001

Note: Data are presented as mean ± SD. PhA = phase angle, VL/H = vector length standardized for height, EnM = Endomorphic Mesomorph, BM = Balanced Mesomorph, EcM = Ectomorphic Mesomorph, M-Ec = Mesomorph Ectomorph, MEc = Mesomorphic Ectomorph, BEc = Balanced Ectomorph. * Results of the one-way ANOVA considering the athletes as a whole sample; ^1^ Different (*p* < 0.008) from Q1; ^2^ Different (*p* < 0.008) from Q2; ^3^ Different (*p* < 0.008) from Q3; ^4^ Different (*p* < 0.008) from Q4.
